# Disease-specific recommendation for rehabilitation: perspective from neurodegenerative diseases

**DOI:** 10.1097/MS9.0000000000000157

**Published:** 2023-01-18

**Authors:** Ikramul Hasan, Md. Selim Reza, Syed S. Haider, M. Emdadul Haque, Talha B. Emran

**Affiliations:** a Department of Pharmaceutical Technology, Faculty of Pharmacy, University of Dhaka; b Department of Pharmacy, BGC Trust University Bangladesh, Chittagong, Bangladesh; c Department of Pharmacy, Faculty of Allied Health Sciences, Daffodil International University, Dhaka, Bangladesh; d Department of Biochemistry and Molecular Biology, UAE University, College of Medicine and Health Sciences, Abu Dhabi, United Arab Emirates

*Dear Editor*,

Rehabilitation is defined by the WHO as ‘a set of interventions designed to optimize functioning and reduce disability in individuals with health conditions in interaction with their environment’^1^,^2^. The health conditions causing disability and compromised functioning can be chronic disease, injuries, trauma, or even aging. The universal health coverage (UHC) target of the sustainable development goals included ‘rehabilitation’ as an essential health service^3^. Properly designed rehabilitation services can improve the quality of life which ultimately will be a very useful tool to achieve sustainable development goal. However, in the low- and middle-income countries like Bangladesh, inequity is present in every aspect of rehabilitation services. A disease-specific rehabilitation service, like rehabilitation for the people with Parkinson, is scared here. In fact, rehabilitation can improve the quality of life of people with Parkinson’s and even might be able to deter the progression of the disease if proper rehabilitation services are given in the early stages of this disease. As Parkinson’s disease is an ailment originated from neurodegeneration, in the primary healthcare facilities the patients are treated by only medication to tackle the neurodegenerative progression^4^. The problem lies in the identification of needs. So, integration of disease-specific recommendation for rehabilitation (DSRR) in the health policy is a must to ensure an effective continuum of care. Again, incorporation of DSRR will also pave the standardized referral pathways for the countries where rehabilitation services are rare in the primary level of healthcare.

## What is disease-specific recommendation for rehabilitation

Every disease has its own prognosis and associated complication. The need for rehabilitation varies according to the disease and condition of the patient. However, we are calling DSRR as the protocol of rehabilitation for all possible chronic diseases and the referral pathways for rehabilitation for each disease. For example, in the case of Parkinson’s disease the need for rehabilitation at the early stage it not so severe but properly planned exercise can deter the progression of the disease and at the more sever stages of Parkinson’s disease rehabilitation can improve the quality of life^5^.

## WHO rehabilitation 2030 Call for action: Why Bangladesh needs disease-specific recommendation for rehabilitation?

In responding to the pressing need for rehabilitation services worldwide WHO launched the ‘Rehabilitation 2030’ initiative in February 2017 and raised a ‘Call for action’^6^. In this initiative, ten areas for concentrated attention were identified to fulfill global needs for rehabilitation^7^. In Bangladesh, there is strong political commitment for achieving UHC but specific action or agenda for rehabilitation is absent here^4^. To strengthen rehabilitation program in national and subnational levels incorporation of this agenda is of utmost important. As Bangladesh is improving in its health service the life expectancy is also increasing as a result need for rehabilitation service is increasing. In Bangladesh, government sponsored healthcare facilities are provided in four levels: community level healthcare (in the rural level, provided by trained health worker), primary level healthcare (Upazila level, provided by medical graduate), secondary-level healthcare (district-level provided by medical graduates and specialist), and tertiary-level healthcare (city area, provided by specialist and experts)^4^. Unfortunately, rehabilitation service is only available in the tertiary-level healthcare as a result at the beginning of the treatment the need for rehabilitation is overlooked. DSRR will fill this gap. Again, incorporation of DSRR in the health system will enable general physicians to start the rehabilitation course immediately after diagnosis.

## Disease-specific recommendation for rehabilitation: In the context of Parkinson’s disease

Parkinson’s disease, a neurodegenerative disorder primarily affects the dopaminergic neurons of substantiating region of the brain. As this region of the brain mainly control the movements, Parkinson’s disease always coupled with many forms of physical disabilities^8^. The primary signs of Parkinsonism are visible after 70–80% reduction of dopamine producing cells. With progression of the disease, dopamine further decreases, resulting in increased problems with movement control, muscular stiffness, tremor, mood, etc.^9^. Though it is not a life-threatening condition but it can hamper daily life and can even make dependent for fulfilling daily needs. However, Parkinson’s disease does not have any complete cure but the quality of life can be improved by means of proper blend of medication and rehabilitation^10^. Generalized relaxation techniques like gentle rocking can decrease excessive muscle tension and thereby can improve flexibility and also can improve range of motion for people with stiffness. Again, rhythmic initiation, deliberate turning movements of the extremities and chest, meditation, diaphragmatic breathing, etc. are some other rehabilitation techniques for managing Parkinson’s disease^11^. However, the detail protocol of rehabilitation for the people with Parkinson’s is described by the many organizations related to Parkinson’s disease like Parkinson Society Canada, Parkinson’s Disease Society of the United Kingdom, etc. but these type of organization is absent in low- and middle-income countries, like Bangladesh. As a result, people of Parkinson’s are being deprived of the holistic treatment.

## Recommendations on rehabilitation in health systems: Inclusion of disease-specific recommendation for rehabilitation

Properly structured health strategy is of utmost important for better healthcare services. WHO provided eight recommendations on rehabilitation in health system. In its eight points at least four points have direct relationship with the health system^12^. However, to make the room for rehabilitation service in the health system of a country like Bangladesh, it is important to incorporate the referrals for rehabilitation from the community level health worker. People usually come to the community healthcare facilities at the initial stage of the treatment and as there is no structured rehabilitation recommendation exists, patients only come to know about the need for rehabilitation service when they go to the specialized healthcare facilities and in the later phases of Parkinson’s disease the rehabilitation may not be effective like the initial stages of the disease. Again, it can be a compensation for the absence of multidisciplinary rehabilitation workforce in the primary level of healthcare services. Ensuring a multidisciplinary workforce in all levels of healthcare facilities is a lengthy process in the low- or middle-income countries like Bangladesh. However, if the general physicians or the healthcare workers of the primary level are aware of the disease-specific recommendation of the rehabilitation services the patient should be able to get the required rehabilitation services from the early stages of the disease.

## Establishing intersectoral links by means of disease-specific recommendation for rehabilitation

To ensure UHC, the complete sets of health requirements of every people should be addressed by the health system and service. Again, comorbidity is very common among the chronic and old patient. So it is of utmost important to strengthen the links between all the sectors of healthcare services to meet population needs efficiently and effectively, and without incorporating rehabilitation related pedagogy as a topic in all health education system it is impossible to make rehabilitation service available in the primary healthcare facilities^7^. Again the efforts for better understanding the DSRR will pave the evidence based protocol of the service. In terms of disease-specific rehabilitation there are many variations from country to country. So, a unified attempt for establishing disease-specific rehabilitation service will increase the networks and partnerships in rehabilitation between different countries.

## Provenance and peer review

Not commissioned, externally peer reviewed.

## Ethical approval

This case report has been reported in line with the Case Report (CARE) guidelines.

## Sources of funding

None.

## Authors’ contribution

I.H.: conceptualization, data curation, writing – original draft preparation, writing – reviewing and editing. M.S.R., S.S.H., and M.E.H.: data curation, writing – original draft preparation, writing – reviewing and editing. T.B.E.: writing – reviewing and editing, visualization, supervision.

## Conflicts of interest disclosure

The authors declare that they have no financial conflict of interest with regard to the content of this report.

## Research registration unique identifying number (UIN)

Not applicable.

## Guarantor

Talha B. Emran.
